# Evaluation of an open source method for calculating physical activity in dogs from harness and collar based sensors

**DOI:** 10.1186/s12917-017-1228-8

**Published:** 2017-11-07

**Authors:** C. Westgarth, C. Ladha

**Affiliations:** 1 Institute of Infection and Global Health and Institute of Veterinary Science, University of Liverpool, Leahurst Campus, Chester High Road, Neston, Cheshire CH64 7TE UK; 2 Institute of Neuroscience, Henry Wellcome Building, The Medical School, Newcastle University, Newcastle upon Tyne, NE2 4HH UK

**Keywords:** Dogs, Physical activity, Accelerometer, Harness, Collar, Exercise, Sensor

## Abstract

**Background:**

The ability to make objective measurements of physical activity in dogs has both clinical and research applications. Accelerometers offer a non-intrusive and convenient solution. Of the commercialy available sensors, measurements are commonly given in manufacturer bespoke units and calculated with closed source approaches. Furthermore, the validation studies that exist for such devices are mounting location dependant, not transferable between brands or not suitable for handling modern raw accelerometry type data.

**Methods:**

This paper describes a validation study of *n* = 5 where 4 sensors were placed on each dog; 2 on a harness and 2 on a collar. Each position held two sensors from different manufacturers; Actigraph (which has previously been validated for use on the collar) and VetSens (which provides un-filtered accelerometry data). The aims of the study was to firstly evaluate the performance of an open-design method of converting raw accelerometry data into units that have previously been validated. Secondly, comparison was made between sensors mounted at each location for determining physical activity state.

**Results:**

Once the raw actigraphy data had been processed with the open-design method, results from a 7 day measurement revealed no significant difference in physical activity estimates via a cutpoint approach between the sensor manufacturers. A second finding was a low inter-site variability between the ventral collar and dorsal harness locations (Pearsons r^2^ = 0.96).

**Conclusions:**

Using the open-design methodology, raw, un-filtered data from the VetSens sensors can be compared or pooled with data gathered from Actigraph sensors. The results also provide strong evidence that ventral collar and dorsal harness sites may be used interchangeably. This enables studies to be designed with a larger inclusion criteria (encompassing dogs that are not well suited for wearing an instrumented collar) and ensures high levels of welfare while maintaining measurement validity.

## Background

In the past decade, accelerometers have gained popularity as tools for measuring dogs’ physical activity. They offer low cost, objective measurements and the size of modern sensors does little to hinder everyday movements and behaviour of the subject; they can be worn continuously as concluded in [1]. Amongst other applications, accelerometers have found use in measuring dysfunction or disease [2–4], making unsupervised behaviour observations [5, 6] and forming part of dog-owner communication systems [7].

The majority of commercially available physical activity sensors contain an accelerometer chip. Fundamentally accelerometers measure movement, or more exactly, they afford a sensitive platform to capture any force exerted on them. This makes them particularly good at detecting both cyclic motions (e.g walking) as well as orientation changes, such as posture transitions. In its raw form, the output of any accelerometer contains a timestamped stream of data containing information related to the forces on each of its 3-axis. Internally, this stream may be sampled up-to several hundred times per second, although a study on human data demonstrated sample rates above 50 Hz provided little benefit (even for complex machine learning activities working in the frequency domain [8]). Even when stationary, accelerometers are sensitive to the reaction forces of gravity. This property allows them to be used effectively used as inclinometers and subsequently good detectors of posture transition and sleep as shown in [9].

When making physical activity measurements the aim is to use the accelerometer chip to detect movements and have the sensor record them for retrospective analysis. For example, in locomotion, the signal measured via the sensor is mostly an attenuated derivative of the reaction force of the thoracic limbs striking the floor. Due to attenuation through skeletal and soft-tissue structures, sensor location may affect the signal. Modelling these attenuations is a complex task is out of scope of this paper. However, from fundamental principles [10] a power spectral analysis will reveal that body accelerations of lower frequencies contain higher energy and will propagate more effectively through bone and soft tissue.

The majority of movements related to physical activity are the result of gross motor functions (e.g. locomotion or posture transitions).For such measurements, it is common to summarise the raw output stream into manageable time-slices of data called epochs (methodology described in [11]). This technique is common practice in human studies and is now also widely adopted for canine measurements [2, 4, 12]. The concept is that a single epoch summary measure contains enough information to make inferences to a coarse physical activity state (over its time period). It is common to give states names such as sedentary, light, moderate or vigorous and each state is associated with a threshold (often termed cutpoint). Depending on the topic of interest, hourly or daily estimates of time in each state can be estimated. Within the field of canine physical activity measurement using accelerometers, current topics of debate are: suitable epoch duration ([4, 12, 13] use 1, 10 and 15 s respectively); best placement location for different types of measurement [12]; and attachment protocol where the current consensus is to use a separate collar for sensor and leash [14, 15].

Although the concept of making summary epoch measures is common, the practicalities of combining a time-slice of 3-axis accelerometer data (which could be hundreds of samples) into a single epoch is complex. Each manufacturer has their own algorithm which will often contain filters to remove non-wear, filter mechanical vibrations and minimise the effect of un-wanted orientation changes (e.g. collar rotations). Subsequently, it is problematic to validate a set of cutpoint thresholds that can be used across device brands. For example, studies such as [16] have used the GT3X (Actigraph Corp, USA) sensor and suggested values which are not directly compatible with [1] which use the Actical (Philips Respironics, Netherlands) sensor. In human studies, an attempt has been made to harmonise across sensor designs by publication of an open methodology for calculating epoch summaries [17]. Using a shaker table resonating at frequencies resembling human movement, this method has been demonstrated to very closely replicate Actigraph Count measures; a unit bespoke to that manufacturer.

For research using animals to be ethical it should minimise discomfort or distress and allow natural behaviour. The latter is particularly important when the purpose of the research is to capture typical behaviour/movement. For the majority of dogs, the collar offers a convenient attachment site for the accelerometer and many studies have reported using it [1, 2, 4, 13, 18]. However, for some breeds (e.g. very small) or a dog with a sensitive throat/neck, the burden of an additional collar (as recommended in [14, 15]) may lead to discomfort and poor quality measurements. In such cases a harness may offer more comfort. As an attachment site, a harness has its own set of idiosyncrasies. Studies such as [6, 7] have reported good compliance with dogs carrying sensors on this site and thus the question arises if measurements made from a collar can be directly compared to those from a harness. Having multiple mounting options and the ability to interchange attachment location within a cohort has the benefit that a wider range of dogs can be recruited without compromising welfare. The ability to use a harness for physical activity measurements also supports the experimentation of combining other sensors (which would be too burdening to be worn on a collar). For example, in humans the addition of GPS data to physical activity has been previously shown to provide context on how environment relates to exercise [19].

The uncertainty as to whether cutpoint values derived for the collar are applicable to the harness originates from the underlying mode of sensor operation; accelerometers are a sensitive platform for measuring forces. For example, a sensor mounted dorsally (e.g. on a harness), has sagittal signal components that reference the angular force between the cervical spine and the crest of the scapula. In contrast, a sensor mounted ventrally (say on a collar) has similar components that reference the rotation of the sternal notch around the dorsal crest of the scapulohumeral joint. While on a skeletal level the forces involved are of the same order of magnitude (if they were not the joint would become unstable), the accelerometer must detect these forces through several layers of fat, muscle and fur. In addition, if the collar is to remain comfortable, it is highly likely that it may move or rotate. Previous work [12] has demonstrated that the attenuation of these signals is sufficient to make estimating walking speed difficult from any other location than a tightly fitting harness.

The first aim of this study is to validate if the open-method for calculating epoch summaries functions on data from dogs. Having a consistent methodology that functions across sensor brands allows for data pooling, prevents brand lock-in and safeguards against scenarios where manufacturers change data processing methodologies without notifying users [20]. Our hypothesis for this part of the study is that the signals originating from a dog and human are sufficiently similar in the frequency domain that the methods developed for humans can be transferred without modification and our two sensors will give similar results.

The second aim of this study is to determine if epoch summary measures obtained from a collar can be compared to those similarly from a harness. The ability to do so will provide flexibility in study design and ensure a consistent level of welfare is maintained through the choice of either a second collar or harness to carry sensors. Our hypothesis is that the coarse nature of the cutpoint methodology will mask effects of mounting specific signal attenuation found to be problematic in approaches such as [12] and thus the findings from each location will be similar.

## Methods

### Experiment design

For this study, *n* = 5 dogs were recruited from a convenience sample belonging to Veterinary School staff members. The sample purposefully included a range of breeds, sizes and ages in order to maximise variability in the captured signal characteristics (see Table 1). The cohort size was chosen to provide sufficient variety in order to test equivalency across a range of movements and is similar to that of a previous validation study [12] (this study had a cohort of *n* = 6). All dogs were deemed healthy by their owners and assessment by the last author. The study was approved by the University of Liverpool Veterinary Ethics Committee.

**Table 1:** Participant information and sensor agreement per dog

Dog	Age (Years)	Breed	Height (cm)	R^2^ VetSens C + H	R^2^ Actigraph C + H	R^2^ Harness Ac + Vs	R^2^ Collar Ac + Vs
A	13	Spaniel cross	48	0.98	0.96	0.97	0.28
B	4	Pug x Chihuahua x Pomeranian	27	0.98	0.97	0.93	0.96
C	6	Patterdale cross	37	0.97	0.97	0.94	0.97
D	0.5	Miniature American Shepherd	36	0.96	0.98	0.95	0.92
E	5	Poodle x Golden retriever	57	0.98	0.97	0.96	0.97

*Ac* Actigraph, *Vs* VetSens, *C* collar, *H* harness

All dogs were selected to wear both an instrumented collar and an instrumented harness. Some had previously worn a harness and for others it was new. Both the collar and harness were fitted with a GT3X (Actigraph Corp, USA) and a VetSens sensor (VetSens UK); a total of four sensors per dog. The Actigraph sensor was chosen as it is a sensor previously validated for canine physical activity measurements and a set of validated cutpoints have been reported [16]. The VetSens sensor was chosen as it compatible with OpenMovement [21] open-source software, provides access to raw un-filtered accelerometer measurements and has had its raw output data verified for equivalency on a shaker table experiment [22]. The sensors were all configured with the same workstation which provided a synchronisation mechanism (to the system time). All the sensors were configured to record continuously for a period of 7 days. The Actigraph sensors automatically measured in Actigraph Count units (the proprietary manufacturer unit) of 60 s epoch duration. The VetSens sensors were set to measure in raw mode with F_s_ = 100 Hz and sensitivity of ±8 g as recommended for capture of canine activity [5].

**Fig. 1:**
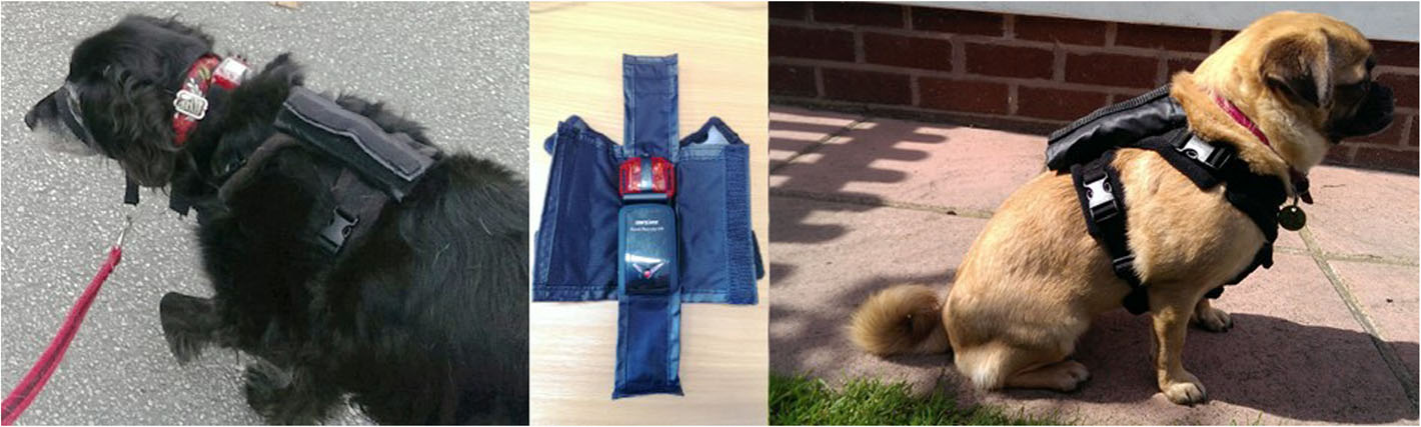
Harness components; a generic compartment housing all sensing equipment and a multisize strap system suitable for different size dogs

The two sensors at each site were physically strapped together using non-stretch tape. The first pair of sensors were attached around the dog neck using a shortened elastic belt and clips supplied with the Actigraph GT3X so that they fit snugly but comfortably to the dog neck (in accordance with recommendations set out in [15]). A dorsal collar position was initially attempted but we found that rotation to the ventral position frequently occurred and efforts to prevent this were abandoned during the capture period. The second pair of sensors were mounted onto a Perfect Fit (Dog Games, UK) three-piece fleece-lined harness using a specially designed pouch integral to the top piece (See Fig. 1).

## Data processing

For convenience and due to their previous validation research, cutpoints suggested by [16] were used as a basis for comparison between sensors. As these figures are specified in manufacturer proprietary units (Actigraph Counts) a prior conversion step was made to the VetSens data such that it was compatible. This conversion step has been openly described and validated in humans [17]. In summary, a 3rd order band-pass Butterworth filter (*f*_*c*_ 0.25–2.5 Hz) is applied to remove DC offset and high frequency noise; such as would be expected from mechanical noise or vibration. The axis are then combined as their Euclidean distance vector into a measure that is reported equivalent in scale to the Actigraph Count.

Measurements from both collar and harness, from each sensor, on each dog, were subsequently summarised into epochs of 60 s. This value was chosen for convenience of processing; it is of similar design to many other studies [1, 23, 24], and the length is sufficient to minimise any synchronisation issues between sensors. Individual epoch summary measures were then first compared across sensor types (within the same location) and then secondly across sites (but within the same sensor type).

### Statistical processing

The resulting data from the two sensors from each location was directly compared; Actigraph-Collar vs VetSens-Collar and Actigraph-Harness vs VetSens-Harness. Correlations were made on time-synchronised epochs of Counts, within each dog, and a mean overall Pearson’s correlation squared (r^2^) value was calculated.

To estimate disagreement between harness-collar for each sensor, a normalised difference is taken against the number of 60 s epochs in 24 h,

$$ disagreement=\left(|{A}_{collar}\hbox{--} {A}_{h arness}|\right)/\left({24}^{\ast }60\right) $$

where A = activity expressed in counts per min and disagreement is in percentage of a 24-h period.

## Results

### Sensor equivalency tests

**Fig. 2:**
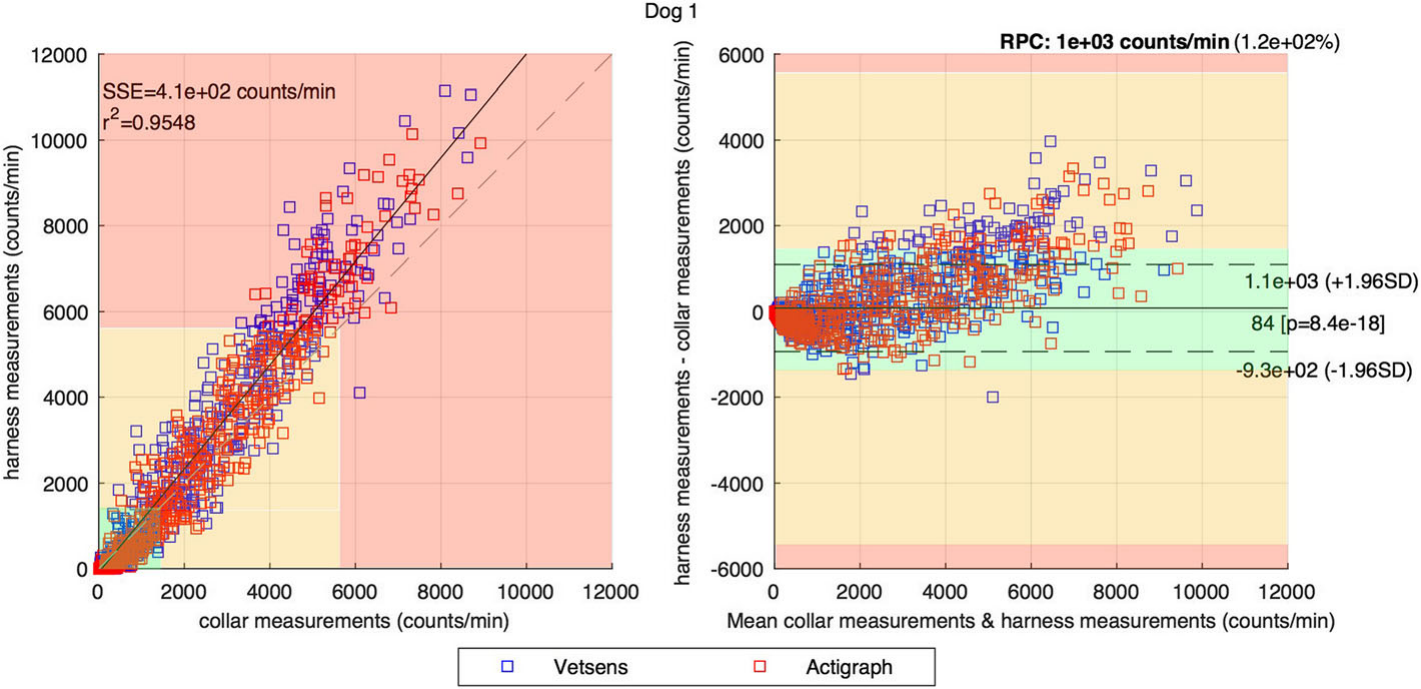
Comparison between harness and collar positions for Vetsens and Actigraph sensors on 1 dog for 24 h. Green, Orange and Red areas mark Sed, Light-Mod and Vig activity levels as defined in [8]

All sensors recorded as configured and a total of 10,080 60s epoch measurements were collected from each sensor (total of 403,200 min over all sensors and dogs). Figure 2 depicts counts from all dogs over a 24 h period and highlights sensor type to illustrate difference. Seven day data from each Actigraph-VetSens sensor pair, on both sites, over all dogs were compared to yield a mean Pearson’s r^2^ = 0.97 and r^2^ = 0.96 for collar and harness location respectively; see Fig. 2. (i). Results per dog are presented in Table 1.

### Collar vs harness position

Applying the cutpoint thresholds from [16] gives results in Table 2. These results represent mean daily averages for all dogs over the 7-day collection period. Reassuringly, the proportion of time spent in Sedentary, Light-Moderate and Vigorous were in line with previous findings for adult dogs [2] (this study reports a 88:10:2 percentage daily time split between Sed:Light-Mod:Vig states). For the VetSens sensor, disagreement between measurements at the harness and collar is 1.2%, 1.5% and 0.3% for Sed, Light-Moderate and Vigorous respectively. For the Actigraph sensor the disagreement between sites is marginally higher at 2.2%, 0.5% 0.4% for Sed, Light-Moderate and Vigorous respectively.

**Table 2:** Mean number of minutes captured by each sensor in each activity state per day

Sensor	Position	S (mins)	LM (mins)	V (mins)
Vetsens	Collar	1154	259	27
	Harness	1171	238	31
Actigraph	Collar	1157	228	37
	Harness	1189	220	31

Activity states determined as per Morrison 2013. N = 5 dogs. (S = Sedentary, LM = Light-Moderate, V = Vigorous)

### Acceptability of the method

With the exception of one (dog C), all dogs tolerated wearing the instrumented harness; for this dog it was removed after 5 days as it was thought it may be becoming uncomfortable. This finding may be incidental as there had not been issues over the first 5 days of the dog wearing it. For the other dogs no adverse effects were reported other than noticing that the dogs were possibly slightly less settled and relaxed in the evenings than they normally would be without the harness. For the very small dog (B), the sensors on the collar were observed to be more intrusive than the harness.

## Discussion

The first aim of this study was to test whether raw data output from the VetSens sensor can be processed using an open-design method to be equivalent to a closed-source equivalent (Actigraph). The results from the intra-site sensor comparisons (VetSens vs Actigraph at the collar and harness locations) was in line with the levels of agreement reported in [15]. Although not perfect, this high degree of correlation provides confidence in replicating the manufacturers bespoke epoch summary units (Actigraph Counts); data gathered from VetSens and Actigraph sensor types can be compared like-for-like basis using previously validated cutpoint sets. Furthermore, data may be pooled with heterogeneous sensor types and analysed together.

The second aim of this study was to evaluate if accelerometer measurements made at the collar could directly be compared to those made at a harness on an epoch-by-epoch basis. Before our experiments, we hypothesised that the epoch summary method would be not be sensitive to the subtle differences in how signal components propagate through the bone and soft-tissue between collar or harness locations. The results from the inter-site comparison (collar vs harness) revealed a high level of agreement between the sites, supporting our hypothesis. Were this level of disagreement translated into a time (0.3% of the day in minutes equates to ~4mins), it would be an order of magnitude below what is considered a reasonable time to spend exercising (~40mins). This result gives confidence in the interchangeability between sites using this technique. Furthermore, this result was consistent across all sizes of dog in our cohort. Our findings are in contrast to previous work [12] that suggested non-compatibility, however the purpose of that study was to determine walking speed (rather than physical activity intensity) and used a much shorter epoch duration (1 s) with only 3 min of recorded data per participant (*n* = 6).

A review into some of the disagreements pointed towards possible root causes of collar rotation or looseness; the harness has the advantage of being unable to rotate. This stresses the importance of consistent and proper mounting protocol and a separate collar for attaching the leash whilst walking. A review of time-synchronised raw signal components from each site was also done and revealed significantly different signal characteristics; predominantly phase and frequency. This leads us to hypothesize that while the relatively coarse approach of constructing epoch summary measures to estimate physical activity will transfer between sites, methods making use of more complex signal features or time-frequency components (such as those in [5, 6]) would not transfer well.

The number of dogs used in this study was relatively small and thus could be considered a limitation; no meaningful inter-dog comparisons could be made for confounding factors such as age, breed, size, weight. However, the scope of this study was limited to equivalency evaluation and correlations were made on an intra-dog basis. The dogs recruited were specifically chosen to provide variability in both physical activity intensity as well as time in spent in each light, moderate and vigorous physical activity states. None of the sampled dogs appeared as an outlier and a long duration was collected to provide optimal chance of all natural behaviour types to be displayed with multiple occurrences (7 continuous days of data on 5 dogs equates to over 400,000 epochs correlation points).

## Conclusions

An open-design methodology for gathering canine physical activity data was evaluated for replication of closed source epoch measurements in manufacturer-specific units. Strong correlation demonstrates its effectiveness and provides the confidence to move towards consistent and validated methodologies of handling raw-data streams; a format sensor manufacturers are trending towards. The open-method also may allow pooling of previously gathered raw accelerometer data across different sensor platforms.

The study also demonstrated equivalency between data gathered from a sensor mounted a harness and a sensor mounted on a collar. Put simply, for the sensors types in this study, cutpoint thresholds validated for the collar can be confidently applied to epoch measurements gathered at the harness. This finding enables studies to be designed with more flexible inclusion criteria enabling measurement of dogs better suited to wearing a harness or use of a harness for reasons such as carrying additional sensors. Being able to interchange collar and harness within a cohort also ensures that consistent levels of welfare can be sought while retaining measurement validity.
